# (Phen­yl)(3-phenyl­sulfonyl-1,2-dihydro­pyrrolo­[1,2-*a*]quinoxalin-1-yl)methanone

**DOI:** 10.1107/S1600536811040335

**Published:** 2011-10-08

**Authors:** Yaşar Dürüst, Akın Sağırlı, Frank R. Fronczek

**Affiliations:** a Department of Chemistry, Abant Izzet Baysal University, TR-14280 Bolu, Turkey; b Department of Chemistry, Louisiana State University, Baton Rouge, LA 70803-1804, USA

## Abstract

In the title mol­ecule, C_24_H_18_N_2_O_3_S, the 13-atom ring system comprising the quinoxaline and fused five-membered ring exhibits an r.m.s. deviation from coplanarity of 0.039 Å, with a maximum deviation of 0.0710 (10) Å for the PhCO-bearing C atom of the five-membered ring. The 10-membered C_8_N_2_ quinoxaline ring system has an r.m.s. deviation from coplanarity of 0.022 Å, with a maximum deviation of 0.0403 (9) Å for the C atom involved in the C=C bond in the five-membered ring. The three atoms of the five-membered ring fused to the quinoxaline ring system show deviations of up to 0.118 (2) Å for the PhCO-bearing C atom. C—N bond distances in the quinoxaline ring system of the title mol­ecule deviate from those in unsubstituted quinoxaline. In particular, the two C—N distances to the N atom involved in the five-membered ring are essentially equal, with values of 1.3786 (17) and 1.3773 (16) Å, unlike the difference of nearly 0.06 Å in quinoxaline.

## Related literature

For the transformation of benzimidazoles into pyrrolo­quinoxalines, see: Ager *et al.* (1988 ▶); Methcohn (1975 ▶). For the synthesis of condensed pyrazines, see: Cheeseman & Cookson (1979 ▶). For the biological activity of quinoxalines, see: Porter (1984 ▶); He *et al.* (2003 ▶); Kim *et al.* (2004 ▶). For cyclization reactions of quinoxaline derivatives, see: Taylor & Hand (1962 ▶, 1963 ▶); Yadav *et al.* (2008 ▶). For the structure of an analogous compound with COOMe at C9 and C10, see: Hirano *et al.* (2002 ▶). For polymorphs of quinoxaline, see: Ranganathan *et al.* (2010 ▶); Anthony *et al.* (1998 ▶). For a description of the Cambridge Structural Database, see: Allen (2002 ▶).
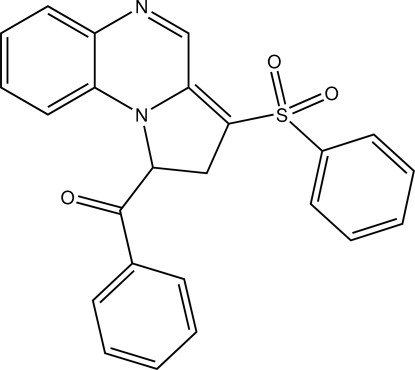


## Experimental

### 

#### Crystal data

C_24_H_18_N_2_O_3_S
*M* *_r_* = 414.46
Monoclinic, 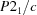

*a* = 19.0915 (9) Å
*b* = 9.9636 (5) Å
*c* = 10.4203 (5) Å
β = 104.6190 (13)°
*V* = 1917.98 (16) Å^3^
*Z* = 4
Cu *K*α radiation
μ = 1.75 mm^−1^
*T* = 90 K
0.30 × 0.27 × 0.13 mm

#### Data collection

Bruker APEXII CCD diffractometer
Absorption correction: multi-scan (*SADABS*; Sheldrick, 2004 ▶) *T*_min_ = 0.622, *T*_max_ = 0.804
31213 measured reflections
3621 independent reflections
3543 reflections with *I* > 2σ(*I*)
*R*_int_ = 0.032

#### Refinement

*R*[*F* ^2^ > 2σ(*F* ^2^)] = 0.029
*wR*(*F* ^2^) = 0.076
*S* = 1.04
3621 reflections
272 parameters
H-atom parameters constrained
Δρ_max_ = 0.41 e Å^−3^
Δρ_min_ = −0.39 e Å^−3^

### 

Data collection: *APEX2* (Bruker, 2007 ▶); cell refinement: *SAINT* (Bruker, 2007 ▶); data reduction: *SAINT*; program(s) used to solve structure: *SHELXTL* (Sheldrick, 2008 ▶); program(s) used to refine structure: *SHELXTL*; molecular graphics: *ORTEP-3 for Windows* (Farrugia, 1997 ▶); software used to prepare material for publication: *SHELXTL*.

## Supplementary Material

Crystal structure: contains datablock(s) global, I. DOI: 10.1107/S1600536811040335/nk2116sup1.cif

Structure factors: contains datablock(s) I. DOI: 10.1107/S1600536811040335/nk2116Isup2.hkl

Supplementary material file. DOI: 10.1107/S1600536811040335/nk2116Isup3.cml

Additional supplementary materials:  crystallographic information; 3D view; checkCIF report

## Figures and Tables

**Fig. 1.**
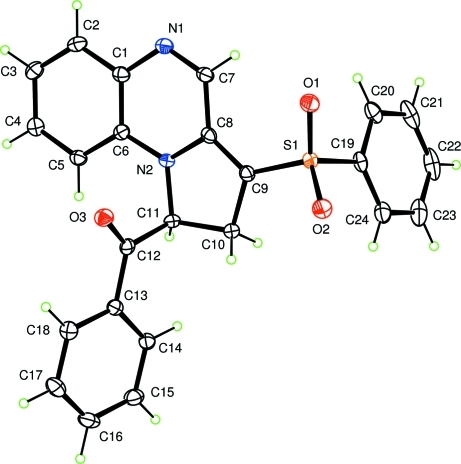
Ellipsoids at the 50% level, with H atoms having arbitrary radius.

## supplementary crystallographic information

### Comment

Despite the fact that pyrrolo[1,2-*a*]quinoxalines have valuable characteristics, in particular, marked biological activity, very limited publications regarding their synthesis have appeared in comparison with similar heterocyles (Cheeseman & Cookson, 1979). One of the most widespread and most widely used methods for the synthesis of pyrrolo-[1,2-*a*]quinoxalines involves the intramolecular cyclization of derivatives of quinoxaline with substituents at position 2, containing at least three carbon atoms with reaction centers capable of nucleophilic attack (Taylor & Hand, 1962; 1963). The benzimidazoles are also transformed into pyrroloquinoxalines by the action of acetylenecarboxylic acid derivatives (Methcohn, 1975; Ager *et al.*, 1988). It is also well known that nitrogen-containing heterocycles are abundant in nature and exhibit diverse and important biological properties (Porter, 1984). While rarely found in nature, quinoxalines find important applications in the pharmaceutical industry and have been shown to possess a broad spectrum of biological activity, including antiviral and antibacterial properties and also act as kinase inhibitors (He *et al.*, 2003; Kim *et al.*, 2004). These heterocyclic ring systems are most commonly assembled by the annulation of a heterocyclic ring onto a pre-existing benzene ring (Yadav *et al.*, 2008). In continuation of our chemistry related to the 1,3-dipolar cycloaddition of heterocyclic N-ylides to electron-deficient alkenes, we have prepared phenylsulfonyl substituted-1,2-dihydropyrrolo[1,2-*a*]quinoxalin-1-ylmethanone by the reaction of *in situ*-generated quinoxalinium ylide and phenyl vinyl sulfone and determined its crystal structure.

A search of the Cambridge Structural Database (version 5.32, Nov. 2010 with May 2011 update, Allen, 2002) yielded only one previous report of a crystal structure (Hirano *et al.*, 2002) containing the 13-atom C_11_N_2_ ring system. It has COOMe groups at C9 and C10 and is unsubstituted at C11, but its coordinates were not deposited. The structures of two polymorphs of unsubstituted quinoxaline have been reported (Anthony *et al.*, 1998; Ranganathan *et al.*, 2010). It is of interest to note the changes to the geometry of the C_4_N_2_ heterocyclic ring of quinoxaline brought about by its fusion to the five-membered ring of the present compound. In quinoxaline, there is considerable double-bond localization into the C═N bonds analogous to C7═N1 and C8═N2. Those bonds have a mean distance of 1.314 Å, averaged over 12 values with a range of values 1.299 - 1.329 Å in the two polymorphs. This is shorter than the mean (of 12) value of 1.371 Å for the bonds corresponding to C1–N1 (range 1.353 - 1.392 Å). The mean distance for the bond analogous to C7–C8 in quinoxaline is 1.406 Å, range 1.373 - 1.421 Å. In the title compound, N1–C1, 1.4007 (17) Å is longer than N1–C7, 1.2904 (18) by an amount greater than in quinoxaline. Also unlike quinoxaline, the two endocyclic bonds to N2 in the title compound are equal, 1.3786 (17) and 1.3773 (16) Å., and C7–C8 is elongated to 1.4536 (17) Å. This is accompanied by a C8═C9 double-bond distance of 1.3555 (18) Å.

The 13-atom ring system C1 through C11, N1 and N2 exhibits an r.m.s. deviation of 0.039 Å, the largest deviations being in the 5-membered ring, 0.0614 (10) Å for C9 and 0.0710 (10) Å for C11. The r.m.s. deviation from coplanarity of the 10-atom quinoxaline fragment C1 through C8, N1 and N2 is only 0.022 Å, with C10 also lying in that plane; +0.002 (2) Å deviation, and C9 and C11 lying farther out of plane, -0.104 (2) and -0.118 (2) Å, respectively.

### Experimental

The quinoxalinium salt (1 mmol, 329 mg), obtained from phenacyl bromide and quinoxaline in acetone, was suspended in dichloromethane (10 ml) and then phenylvinylsulfone (1 mmol, 168 mg) was added. Under vigorous stirring, triethylamine (1.4 ml, 1 mmol) was added dropwise. The progress of the reaction was monitored by TLC. After 20 min the reaction mixture was washed with water (2 *X* 10 ml) and solvent was evaporated. The residual crude product was purified with column chromatography using hexane-ethyl acetate as eluent. The cycloaddition product was recrystallized from CDCl_3_ to give orange crystals. *M*.p.148–150°C. *R*_f_: 0.65 (ethyl acetate-n-hexane; 1:1). IR (KBr): ν= 1696 (C═O),1594,1566 1479, 1300, 1223, 1149, 1078, 719, 611 cm^-1. 1^H NMR (400 MHz, CDCl_3_): δ = 9.11 (s, 1H), 7.92 (d, J =7.2 Hz, 2H), 7.86 (d, J =7.6 Hz, 2H), 7.69–7.65 (q, 2H), 7.56–7.47 (m, 5H), 7.36 (s, 1H), 7.21 (t, 1H), 7.06 (t, 1H), 6.30 (d, J = 8.0 Hz, 1H), 5.84–5.79 (dd, J = 14.0, 6.4 Hz, 1H), 3.56 (t, J = 16.0 Hz, 1H), 3.00–2.95 (dd, J = 14.6, 6.0 Hz, 1H). ^13^C NMR (100 MHz, DMSO-*d_6_*): δ = 193.3 (C═O), 145.1, 142.7, 142.5, 135.2, 135.0, 133.4, 132.7, 132.4, 132.3, 130.2, 129.8, 129.8, 126.4, 123.1, 113.6, 99.1, 63.7, 33.9. LC—MS (70 eV): (*m/z*, %)= 415.80 (100) [*M*+H]^+^.

### Refinement

H atoms on C were placed in idealized positions with C—H distances 0.95 - 1.00 Å and thereafter treated as riding. *U*_iso_ for H were assigned as 1.2 times *U*_eq_ of the attached atoms.

### Crystal data

**Table:**

C_24_H_18_N_2_O_3_S	*F*(000) = 864
*M**_r_* = 414.46	*D*_x_ = 1.435 Mg m^−^^3^
Monoclinic, *P*2_1_/*c*	Cu *K*α radiation, λ = 1.54178 Å
Hall symbol: -P 2ybc	Cell parameters from 9862 reflections
*a* = 19.0915 (9) Å	θ = 4.4–70.1°
*b* = 9.9636 (5) Å	µ = 1.75 mm^−^^1^
*c* = 10.4203 (5) Å	*T* = 90 K
β = 104.6190 (13)°	Rectangular prism, orange
*V* = 1917.98 (16) Å^3^	0.30 × 0.27 × 0.13 mm
*Z* = 4	

### Data collection

**Table:**

Bruker APEXII CCD diffractometer	3621 independent reflections
Radiation source: fine-focus sealed tube	3543 reflections with *I* > 2σ(*I*)
graphite	*R*_int_ = 0.032
φ and ω scans	θ_max_ = 70.2°, θ_min_ = 2.4°
Absorption correction: multi-scan (*SADABS*; Sheldrick, 2004)	*h* = −23→23
*T*_min_ = 0.622, *T*_max_ = 0.804	*k* = −10→12
31213 measured reflections	*l* = −12→12

### Refinement

**Table:**

Refinement on *F*^2^	Secondary atom site location: difference Fourier map
Least-squares matrix: full	Hydrogen site location: inferred from neighbouring sites
*R*[*F*^2^ > 2σ(*F*^2^)] = 0.029	H-atom parameters constrained
*wR*(*F*^2^) = 0.076	*w* = 1/[σ^2^(*F*_o_^2^) + (0.0349*P*)^2^ + 1.1954*P*] where *P* = (*F*_o_^2^ + 2*F*_c_^2^)/3
*S* = 1.04	(Δ/σ)_max_ = 0.001
3621 reflections	Δρ_max_ = 0.41 e Å^−^^3^
272 parameters	Δρ_min_ = −0.39 e Å^−^^3^
0 restraints	Extinction correction: *SHELXTL* (Sheldrick, 2008), Fc^*^=kFc[1+0.001xFc^2^λ^3^/sin(2θ)]^-1/4^
Primary atom site location: structure-invariant direct methods	Extinction coefficient: 0.00108 (12)

### Special details

**Table:**

Geometry. All e.s.d.'s (except the e.s.d. in the dihedral angle between two l.s. planes) are estimated using the full covariance matrix. The cell e.s.d.'s are taken into account individually in the estimation of e.s.d.'s in distances, angles and torsion angles; correlations between e.s.d.'s in cell parameters are only used when they are defined by crystal symmetry. An approximate (isotropic) treatment of cell e.s.d.'s is used for estimating e.s.d.'s involving l.s. planes.
Refinement. Refinement of *F*^2^ against ALL reflections. The weighted *R*-factor *wR* and goodness of fit *S* are based on *F*^2^, conventional *R*-factors *R* are based on *F*, with *F* set to zero for negative *F*^2^. The threshold expression of *F*^2^ > σ(*F*^2^) is used only for calculating *R*-factors(gt) *etc*. and is not relevant to the choice of reflections for refinement. *R*-factors based on *F*^2^ are statistically about twice as large as those based on *F*, and *R*- factors based on ALL data will be even larger.

### Fractional atomic coordinates and isotropic or equivalent isotropic displacement parameters (Å^2^)

**Table:**

	*x*	*y*	*z*	*U*_iso_*/*U*_eq_	
S1	0.347686 (15)	0.61731 (3)	0.66691 (3)	0.01450 (10)	
O1	0.36497 (5)	0.53406 (10)	0.56612 (9)	0.0205 (2)	
O2	0.31216 (5)	0.74476 (9)	0.63034 (9)	0.0200 (2)	
O3	0.11000 (5)	0.43117 (9)	0.71433 (9)	0.0190 (2)	
N1	0.29905 (6)	0.15898 (11)	0.69500 (10)	0.0166 (2)	
N2	0.24574 (6)	0.35506 (11)	0.83452 (10)	0.0146 (2)	
C1	0.25306 (7)	0.12578 (13)	0.77577 (12)	0.0155 (3)	
C2	0.23431 (7)	−0.00846 (13)	0.78555 (13)	0.0182 (3)	
H2	0.2537	−0.0755	0.7395	0.022*	
C3	0.18766 (7)	−0.04454 (13)	0.86193 (13)	0.0198 (3)	
H3	0.1741	−0.1358	0.8665	0.024*	
C4	0.16049 (7)	0.05308 (13)	0.93218 (13)	0.0181 (3)	
H4	0.1290	0.0275	0.9854	0.022*	
C5	0.17878 (7)	0.18683 (13)	0.92537 (12)	0.0162 (3)	
H5	0.1602	0.2527	0.9739	0.019*	
C6	0.22492 (6)	0.22410 (13)	0.84637 (12)	0.0142 (3)	
C7	0.31442 (7)	0.28371 (13)	0.68290 (12)	0.0159 (3)	
H7	0.3450	0.3057	0.6268	0.019*	
C8	0.28749 (6)	0.39240 (13)	0.75032 (12)	0.0142 (3)	
C9	0.29573 (6)	0.52749 (13)	0.74933 (12)	0.0150 (3)	
C10	0.25941 (7)	0.59237 (13)	0.84700 (13)	0.0170 (3)	
H10A	0.2265	0.6655	0.8048	0.020*	
H10B	0.2955	0.6285	0.9247	0.020*	
C11	0.21643 (7)	0.47295 (12)	0.88718 (12)	0.0145 (3)	
H11	0.2249	0.4671	0.9857	0.017*	
C12	0.13534 (7)	0.48735 (12)	0.81931 (12)	0.0139 (3)	
C13	0.09098 (7)	0.57500 (12)	0.88358 (12)	0.0144 (3)	
C14	0.12184 (7)	0.65545 (13)	0.99308 (12)	0.0163 (3)	
H14	0.1727	0.6549	1.0296	0.020*	
C15	0.07783 (7)	0.73617 (14)	1.04836 (13)	0.0210 (3)	
H15	0.0987	0.7912	1.1226	0.025*	
C16	0.00346 (8)	0.73669 (14)	0.99538 (15)	0.0231 (3)	
H16	−0.0264	0.7924	1.0333	0.028*	
C17	−0.02763 (7)	0.65613 (15)	0.88708 (14)	0.0223 (3)	
H17	−0.0786	0.6563	0.8515	0.027*	
C18	0.01584 (7)	0.57577 (14)	0.83122 (13)	0.0181 (3)	
H18	−0.0054	0.5209	0.7570	0.022*	
C19	0.42916 (7)	0.64997 (14)	0.78853 (12)	0.0168 (3)	
C20	0.48648 (7)	0.56023 (15)	0.80223 (14)	0.0223 (3)	
H20	0.4825	0.4844	0.7456	0.027*	
C21	0.54965 (8)	0.58369 (18)	0.90036 (15)	0.0304 (4)	
H21	0.5896	0.5242	0.9109	0.037*	
C22	0.55422 (8)	0.69423 (19)	0.98282 (14)	0.0326 (4)	
H22	0.5975	0.7099	1.0497	0.039*	
C23	0.49673 (8)	0.78208 (17)	0.96936 (14)	0.0292 (3)	
H23	0.5006	0.8570	1.0272	0.035*	
C24	0.43328 (7)	0.76078 (15)	0.87121 (13)	0.0218 (3)	
H24	0.3936	0.8207	0.8608	0.026*	

### Atomic displacement parameters (Å^2^)

**Table:**

	*U*^11^	*U*^22^	*U*^33^	*U*^12^	*U*^13^	*U*^23^
S1	0.01365 (16)	0.01546 (17)	0.01436 (16)	−0.00031 (11)	0.00346 (11)	0.00024 (11)
O1	0.0224 (5)	0.0226 (5)	0.0183 (5)	−0.0015 (4)	0.0087 (4)	−0.0023 (4)
O2	0.0200 (5)	0.0191 (5)	0.0203 (5)	0.0021 (4)	0.0039 (4)	0.0038 (4)
O3	0.0197 (5)	0.0201 (5)	0.0166 (4)	0.0001 (4)	0.0035 (4)	−0.0032 (4)
N1	0.0158 (5)	0.0174 (6)	0.0162 (5)	0.0023 (4)	0.0034 (4)	−0.0019 (4)
N2	0.0142 (5)	0.0138 (5)	0.0169 (5)	0.0011 (4)	0.0060 (4)	−0.0022 (4)
C1	0.0137 (6)	0.0174 (7)	0.0142 (6)	0.0026 (5)	0.0014 (5)	−0.0007 (5)
C2	0.0204 (6)	0.0151 (6)	0.0179 (6)	0.0039 (5)	0.0025 (5)	−0.0022 (5)
C3	0.0235 (7)	0.0136 (6)	0.0210 (7)	0.0000 (5)	0.0034 (5)	0.0012 (5)
C4	0.0172 (6)	0.0191 (7)	0.0175 (6)	−0.0001 (5)	0.0033 (5)	0.0024 (5)
C5	0.0151 (6)	0.0167 (6)	0.0166 (6)	0.0022 (5)	0.0037 (5)	−0.0008 (5)
C6	0.0125 (6)	0.0143 (6)	0.0141 (6)	0.0012 (5)	−0.0002 (5)	−0.0005 (5)
C7	0.0139 (6)	0.0185 (7)	0.0156 (6)	0.0018 (5)	0.0043 (5)	−0.0024 (5)
C8	0.0103 (5)	0.0180 (6)	0.0134 (6)	0.0004 (5)	0.0015 (4)	−0.0010 (5)
C9	0.0124 (6)	0.0166 (6)	0.0158 (6)	−0.0003 (5)	0.0033 (5)	−0.0016 (5)
C10	0.0164 (6)	0.0144 (6)	0.0218 (6)	−0.0009 (5)	0.0077 (5)	−0.0034 (5)
C11	0.0158 (6)	0.0125 (6)	0.0159 (6)	0.0016 (5)	0.0051 (5)	−0.0024 (5)
C12	0.0165 (6)	0.0113 (6)	0.0146 (6)	−0.0010 (5)	0.0052 (5)	0.0024 (5)
C13	0.0162 (6)	0.0126 (6)	0.0155 (6)	0.0013 (5)	0.0060 (5)	0.0033 (5)
C14	0.0155 (6)	0.0153 (6)	0.0185 (6)	0.0003 (5)	0.0047 (5)	0.0008 (5)
C15	0.0233 (7)	0.0187 (7)	0.0212 (6)	0.0012 (5)	0.0062 (5)	−0.0047 (5)
C16	0.0223 (7)	0.0220 (7)	0.0274 (7)	0.0077 (5)	0.0110 (6)	−0.0004 (6)
C17	0.0148 (6)	0.0264 (7)	0.0254 (7)	0.0045 (5)	0.0044 (5)	0.0023 (6)
C18	0.0176 (6)	0.0188 (7)	0.0170 (6)	0.0004 (5)	0.0028 (5)	0.0012 (5)
C19	0.0138 (6)	0.0215 (7)	0.0158 (6)	−0.0034 (5)	0.0049 (5)	0.0039 (5)
C20	0.0181 (6)	0.0270 (7)	0.0240 (7)	0.0009 (5)	0.0094 (5)	0.0079 (6)
C21	0.0140 (6)	0.0467 (10)	0.0311 (8)	0.0020 (6)	0.0065 (6)	0.0193 (7)
C22	0.0188 (7)	0.0560 (11)	0.0203 (7)	−0.0147 (7)	−0.0002 (5)	0.0121 (7)
C23	0.0287 (8)	0.0396 (9)	0.0196 (7)	−0.0167 (7)	0.0063 (6)	−0.0021 (6)
C24	0.0205 (7)	0.0253 (7)	0.0206 (6)	−0.0059 (5)	0.0070 (5)	−0.0001 (5)

### Geometric parameters (Å, °)

**Table:**

S1—O1	1.4404 (9)	C10—H10B	0.9900
S1—O2	1.4448 (10)	C11—C12	1.5377 (17)
S1—C9	1.7183 (13)	C11—H11	1.0000
S1—C19	1.7710 (13)	C12—C13	1.4882 (17)
O3—C12	1.2152 (16)	C13—C14	1.3972 (18)
N1—C7	1.2904 (18)	C13—C18	1.4003 (18)
N1—C1	1.4007 (17)	C14—C15	1.3889 (18)
N2—C8	1.3773 (16)	C14—H14	0.9500
N2—C6	1.3786 (17)	C15—C16	1.387 (2)
N2—C11	1.4660 (15)	C15—H15	0.9500
C1—C2	1.3950 (19)	C16—C17	1.390 (2)
C1—C6	1.4103 (17)	C16—H16	0.9500
C2—C3	1.3833 (19)	C17—C18	1.3829 (19)
C2—H2	0.9500	C17—H17	0.9500
C3—C4	1.3937 (19)	C18—H18	0.9500
C3—H3	0.9500	C19—C20	1.3924 (19)
C4—C5	1.3840 (19)	C19—C24	1.390 (2)
C4—H4	0.9500	C20—C21	1.390 (2)
C5—C6	1.3991 (18)	C20—H20	0.9500
C5—H5	0.9500	C21—C22	1.386 (3)
C7—C8	1.4536 (17)	C21—H21	0.9500
C7—H7	0.9500	C22—C23	1.383 (2)
C8—C9	1.3555 (18)	C22—H22	0.9500
C9—C10	1.5135 (17)	C23—C24	1.390 (2)
C10—C11	1.5613 (17)	C23—H23	0.9500
C10—H10A	0.9900	C24—H24	0.9500
			
O1—S1—O2	119.51 (6)	N2—C11—C10	103.55 (9)
O1—S1—C9	109.38 (6)	C12—C11—C10	109.99 (10)
O2—S1—C9	107.32 (6)	N2—C11—H11	111.1
O1—S1—C19	107.83 (6)	C12—C11—H11	111.1
O2—S1—C19	107.24 (6)	C10—C11—H11	111.1
C9—S1—C19	104.57 (6)	O3—C12—C13	122.20 (11)
C7—N1—C1	118.60 (11)	O3—C12—C11	119.79 (11)
C8—N2—C6	122.62 (11)	C13—C12—C11	117.97 (10)
C8—N2—C11	111.04 (10)	C14—C13—C18	119.63 (12)
C6—N2—C11	125.38 (10)	C14—C13—C12	122.25 (11)
C2—C1—N1	118.80 (11)	C18—C13—C12	118.12 (11)
C2—C1—C6	119.30 (12)	C15—C14—C13	119.77 (12)
N1—C1—C6	121.90 (12)	C15—C14—H14	120.1
C3—C2—C1	120.36 (12)	C13—C14—H14	120.1
C3—C2—H2	119.8	C14—C15—C16	120.17 (13)
C1—C2—H2	119.8	C14—C15—H15	119.9
C2—C3—C4	119.99 (12)	C16—C15—H15	119.9
C2—C3—H3	120.0	C17—C16—C15	120.35 (12)
C4—C3—H3	120.0	C17—C16—H16	119.8
C5—C4—C3	120.85 (12)	C15—C16—H16	119.8
C5—C4—H4	119.6	C18—C17—C16	119.83 (12)
C3—C4—H4	119.6	C18—C17—H17	120.1
C4—C5—C6	119.35 (12)	C16—C17—H17	120.1
C4—C5—H5	120.3	C17—C18—C13	120.25 (12)
C6—C5—H5	120.3	C17—C18—H18	119.9
N2—C6—C5	122.85 (11)	C13—C18—H18	119.9
N2—C6—C1	117.02 (11)	C20—C19—C24	121.75 (13)
C5—C6—C1	120.12 (12)	C20—C19—S1	118.75 (11)
N1—C7—C8	123.60 (12)	C24—C19—S1	119.44 (10)
N1—C7—H7	118.2	C21—C20—C19	118.75 (14)
C8—C7—H7	118.2	C21—C20—H20	120.6
C9—C8—N2	111.10 (11)	C19—C20—H20	120.6
C9—C8—C7	132.87 (12)	C22—C21—C20	119.79 (14)
N2—C8—C7	116.03 (11)	C22—C21—H21	120.1
C8—C9—C10	110.20 (11)	C20—C21—H21	120.1
C8—C9—S1	127.13 (10)	C23—C22—C21	121.06 (13)
C10—C9—S1	122.27 (9)	C23—C22—H22	119.5
C9—C10—C11	102.46 (10)	C21—C22—H22	119.5
C9—C10—H10A	111.3	C22—C23—C24	119.98 (15)
C11—C10—H10A	111.3	C22—C23—H23	120.0
C9—C10—H10B	111.3	C24—C23—H23	120.0
C11—C10—H10B	111.3	C23—C24—C19	118.67 (14)
H10A—C10—H10B	109.2	C23—C24—H24	120.7
N2—C11—C12	109.74 (10)	C19—C24—H24	120.7
			
C7—N1—C1—C2	177.83 (12)	C8—N2—C11—C12	105.72 (11)
C7—N1—C1—C6	−1.70 (18)	C6—N2—C11—C12	−63.24 (15)
N1—C1—C2—C3	−178.48 (11)	C8—N2—C11—C10	−11.67 (13)
C6—C1—C2—C3	1.06 (19)	C6—N2—C11—C10	179.38 (11)
C1—C2—C3—C4	−1.5 (2)	C9—C10—C11—N2	12.32 (12)
C2—C3—C4—C5	0.9 (2)	C9—C10—C11—C12	−104.89 (11)
C3—C4—C5—C6	0.27 (19)	N2—C11—C12—O3	−19.31 (16)
C8—N2—C6—C5	−175.85 (11)	C10—C11—C12—O3	93.98 (13)
C11—N2—C6—C5	−8.11 (19)	N2—C11—C12—C13	162.91 (10)
C8—N2—C6—C1	4.95 (17)	C10—C11—C12—C13	−83.80 (13)
C11—N2—C6—C1	172.69 (11)	O3—C12—C13—C14	−170.10 (12)
C4—C5—C6—N2	−179.91 (11)	C11—C12—C13—C14	7.62 (17)
C4—C5—C6—C1	−0.74 (18)	O3—C12—C13—C18	9.85 (18)
C2—C1—C6—N2	179.30 (11)	C11—C12—C13—C18	−172.43 (11)
N1—C1—C6—N2	−1.17 (17)	C18—C13—C14—C15	−0.50 (19)
C2—C1—C6—C5	0.08 (18)	C12—C13—C14—C15	179.45 (12)
N1—C1—C6—C5	179.61 (11)	C13—C14—C15—C16	0.2 (2)
C1—N1—C7—C8	1.00 (18)	C14—C15—C16—C17	0.3 (2)
C6—N2—C8—C9	175.24 (11)	C15—C16—C17—C18	−0.5 (2)
C11—N2—C8—C9	5.93 (14)	C16—C17—C18—C13	0.2 (2)
C6—N2—C8—C7	−5.56 (17)	C14—C13—C18—C17	0.30 (19)
C11—N2—C8—C7	−174.87 (10)	C12—C13—C18—C17	−179.65 (12)
N1—C7—C8—C9	−178.52 (13)	O1—S1—C19—C20	−24.23 (12)
N1—C7—C8—N2	2.50 (18)	O2—S1—C19—C20	−154.13 (10)
N2—C8—C9—C10	2.97 (15)	C9—S1—C19—C20	92.12 (11)
C7—C8—C9—C10	−176.05 (13)	O1—S1—C19—C24	158.43 (10)
N2—C8—C9—S1	175.80 (9)	O2—S1—C19—C24	28.53 (12)
C7—C8—C9—S1	−3.2 (2)	C9—S1—C19—C24	−85.22 (11)
O1—S1—C9—C8	17.24 (14)	C24—C19—C20—C21	−0.8 (2)
O2—S1—C9—C8	148.29 (12)	S1—C19—C20—C21	−178.08 (10)
C19—S1—C9—C8	−98.03 (12)	C19—C20—C21—C22	0.6 (2)
O1—S1—C9—C10	−170.72 (10)	C20—C21—C22—C23	0.1 (2)
O2—S1—C9—C10	−39.67 (11)	C21—C22—C23—C24	−0.5 (2)
C19—S1—C9—C10	74.02 (11)	C22—C23—C24—C19	0.3 (2)
C8—C9—C10—C11	−9.76 (13)	C20—C19—C24—C23	0.4 (2)
S1—C9—C10—C11	176.99 (9)	S1—C19—C24—C23	177.63 (10)
